# Superior vena cava syndrome after pacemaker implantation treated with direct oral anticoagulation

**DOI:** 10.1186/s12959-021-00321-7

**Published:** 2021-11-08

**Authors:** Nicola Mumoli, Antonino Mazzone, Isabella Evangelista, Marco Cei, Alessandra Colombo

**Affiliations:** Department of Internal Medicine,, Ospedale Fornaroli, via Donatori Sangue, 50, 20013 Magenta, MI Italy

**Keywords:** Superior vena cava, Pacemaker, Thrombosis

## Abstract

**Background:**

Superior Vena Cava (SVC) syndrome, is a quite rare but serious complication after pacemaker lead implantation; most patients are asymptomatic due to the development of adequate venous collateral circulation.

**Case presentation:**

We report a case of a 75-year-old woman who developed SVC syndrome after transvenous pacemaker implantation with complete resolution of the thrombosis after 3 months of oral anticoagulation.

**Conclusions:**

**Generally** other causes as malignancy are considered to be the most common etiology of SVC syndrome, but benign iatrogenic causes, mainly intravascular devices (central vein catheters, cardiac defibrillators and pacemaker wires), are becoming increasingly common. Procedures performed on venous vasculature, causing a possible intimal injury or vein stenosis, provoked by transvenous leads, seem to be the most reasonable explanation for the observed complication.

## Background

The Superior Vena Cava (SVC) represents the main drainage vessel for the venous blood of the head, neck, upper extremities, and upper chest. SVC syndrome is a very rare but debilitating complication after pacemaker lead implantation. Symptoms depend on how quickly the obstruction establishes. They include headache, upper limb edema, jugular vein distention, cyanosis, and facial swelling.

## Case presentation

**Fig. 1:**
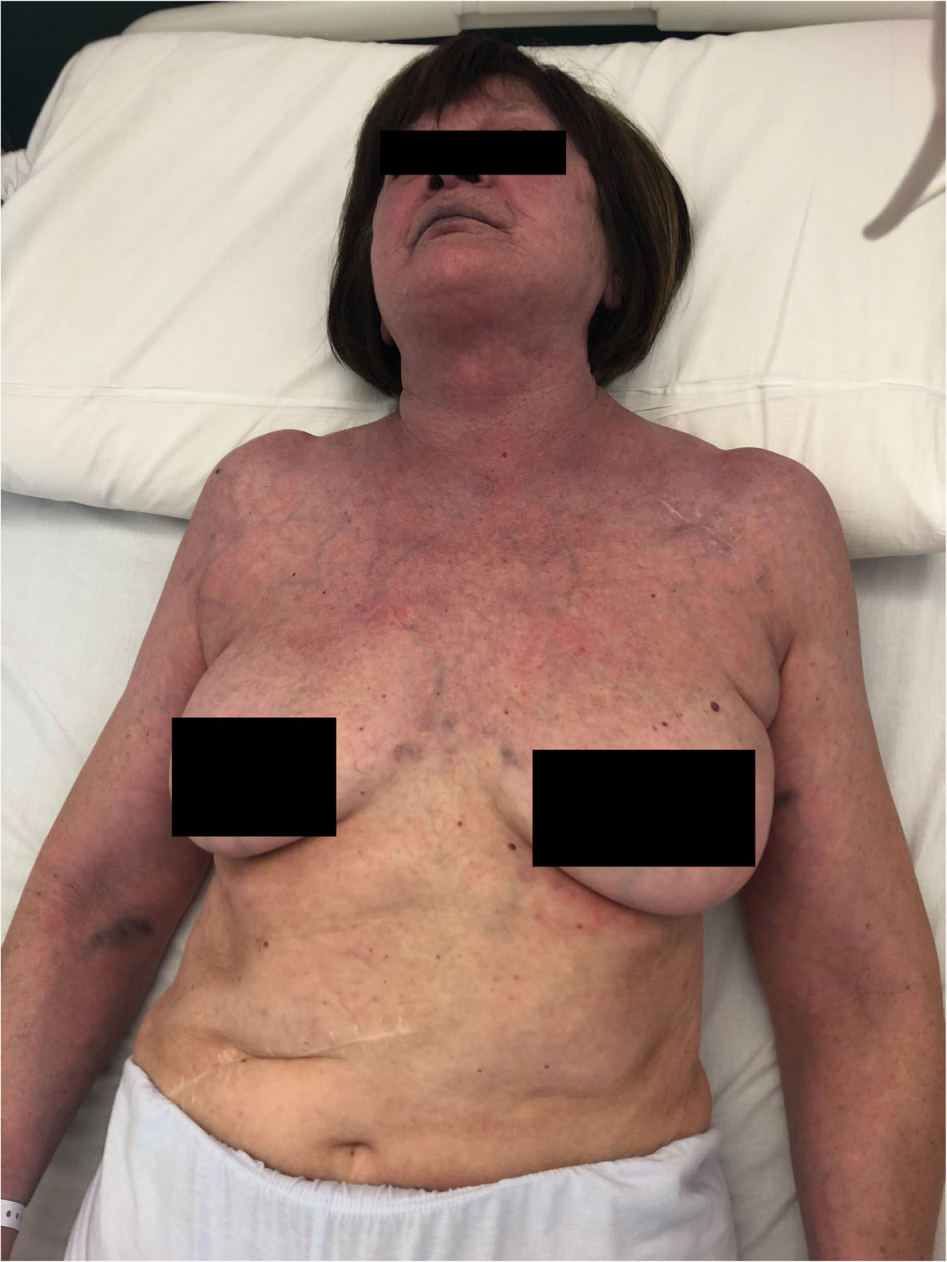
The image show cyanosis, edema and prominent engorged vasculature in the face, neck, bilateral upper limbs and anterior chest wall

**Fig. 2:**
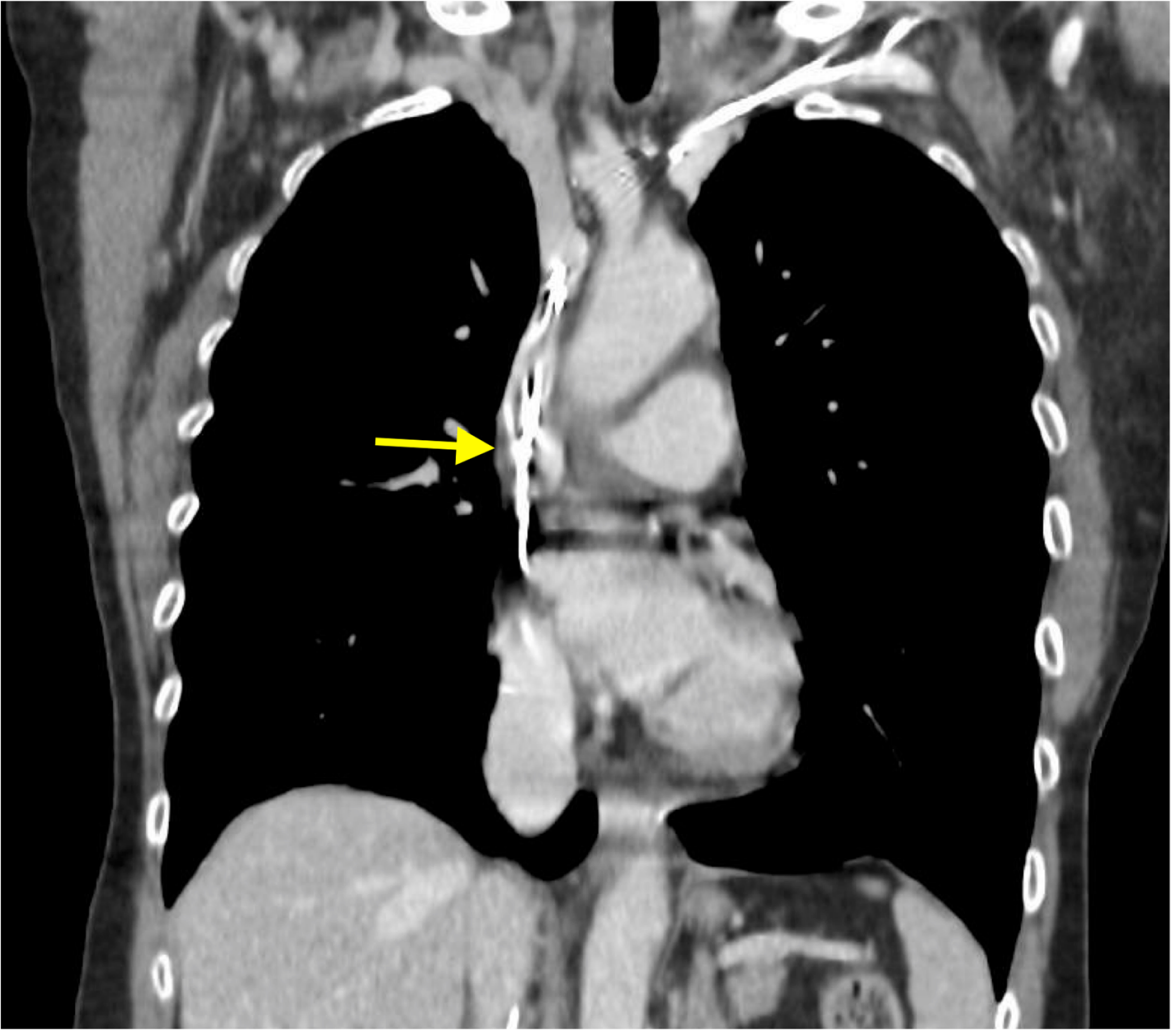
Thoracic CT angiography in coronal view, showing a superior vena cava obstruction determined by thrombus (arrow) around indwelling leads

**Fig. 3:**
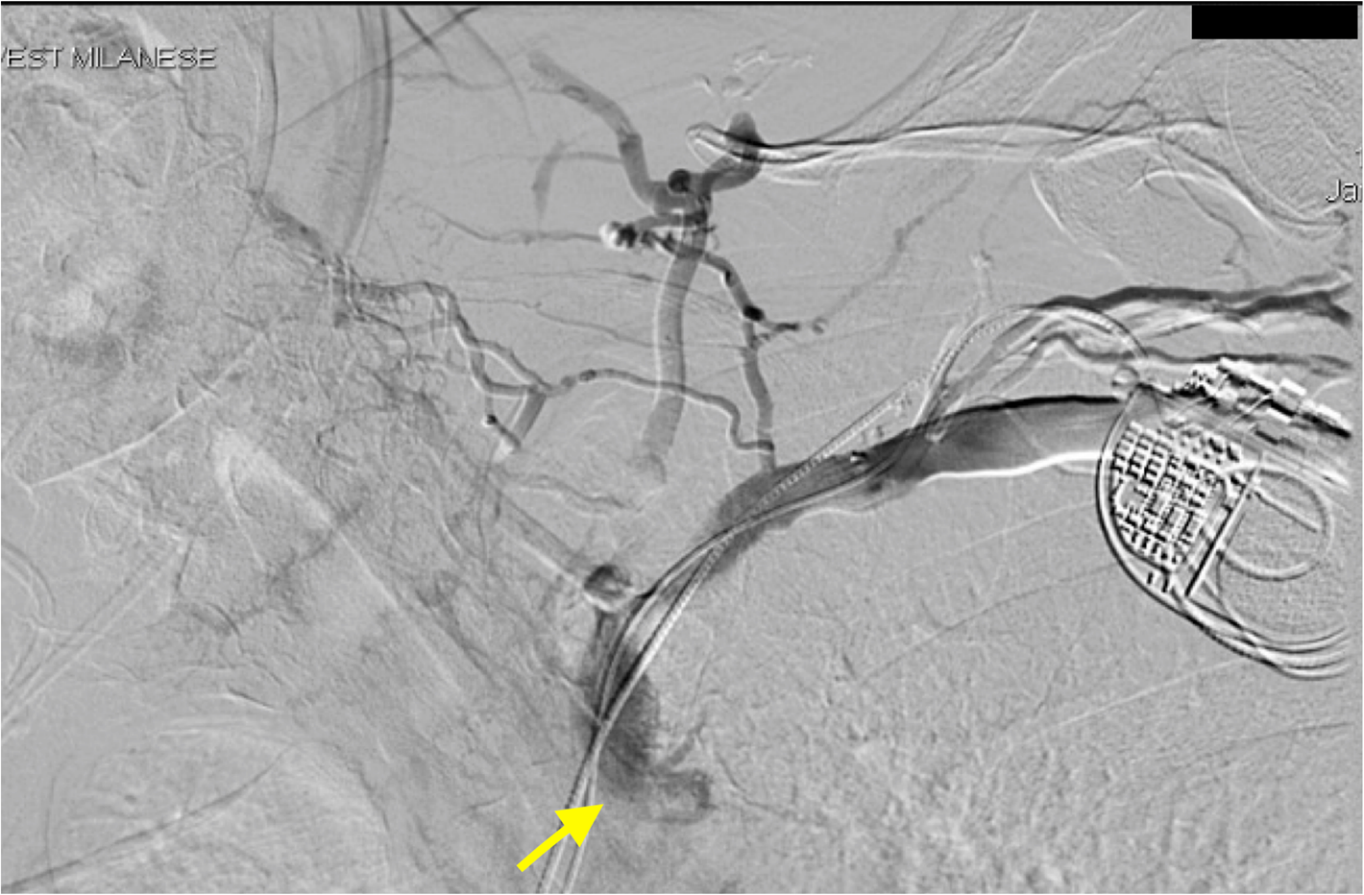
superior cavography showing a minus image in superior vena cava (arrow) around indwelling leads, with increased flow through the collateral circulation (indirect thrombus demonstration)

A 75-year-old woman was admitted to our Emergency Department because of headache and progressive cyanosis and swelling of the face, neck, thorax, and both upper extremities. She denied any pain, but reported heaviness of both arms. These symptoms worsened gradually over the past 4 months. Her medical history included pacemaker implantation 2 years before for sick sinus syndrome; physical examination revealed cyanosis, edema, and prominent engorged vasculature on the face, neck, bilateral upper limbs and anterior chest wall (Fig. 1). Complete blood count, coagulation, renal and hepatic function were within normal limits, except for an increase in D-dimer value (925 ng/mL; upper reference limit, < 270 ng/mL). Computed tomography (CT) angiography of the chest (Fig. 2, arrow) and superior cavography (Fig. 3, arrow) revealed a thrombus obstructing the superior vena cava around indwelling pacemaker leads, with increased flow through the collateral circulation. Balloon angioplasty was considered [1] but the patient refused. Due to the long period of onset of symptoms, treatment with fibrinolytics was not considered appropriate. After a week of full dose subcutaneous enoxaparin, anticoagulation with Edoxaban 60 mg once daily was started and gradually a complete resolution of the symptoms was obtained. The patient was discharged in a stable condition 10 days later.

Three months later, the patient was free of symptoms and a chest CT angiography revealed complete resolution of the thrombosis and the anticoagulant was interrupted without evidence of recurrence 3 months afterward.

## Discussion and conclusions

Superior Vena Cava (SVC) syndrome [2, 3] is a very rare but debilitating complication after pacemaker lead implantation. Symptoms depend on how quickly the obstruction establishes; however, the insurgence of thrombosis caused by pacemaker leads seems to be unrelated to the time elapsed from the procedure [4]. Most patients are often less symptomatic due to the development of collateral circulation. Several causes lead to this syndrome. The most common is malignancy (85%): lung cancer, lymphomas; metastasis to the mediastinum from breast cancer or gastrointestinal tumors, primary mediastinal tumors. The mechanisms most involved are extrinsic compression and neoplastic infiltration of SVC. Less commonly, non-oncologic causes may occur: infections, spontaneous thrombosis, and iatrogenic causes. Among the latter, radiotherapy on the mediastinum and thrombosis or infections of intravascular devices (central vein catheters, cardiac defibrillators, and pacemaker wires) are becoming increasingly common. However, in a large series from Rice [5], a pacemaker was considered to be the cause of SVC syndrome in only 1 out of 78 cases (1.28%). Procedures performed on venous vasculature, causing a possible intimal injury or vein stenosis, provoked by transvenous leads, seem to be the most reasonable explanation for the observed complication. The treatment of SVC syndrome involves the use of medical, interventional, or surgical therapy. Medical management includes anticoagulants or thrombolytics; interventional procedures commonly performed include balloon angioplasty and stenting. The duration of symptoms before the onset of thrombolytic therapy, can often guide the most appropriate approach. The success rate of thrombolytic therapy is greater if treatment is begun less than or equal to 5 days after the symptoms started. Endovascular repair is less invasive but equally effective compared to the surgical approach. The open repair treatment is mostly used in SVC syndrome due to mediastinal fibrosis [2]. When the cause is pacemaker implantation two treatments are possible. The first one is the lead removal, stent implantation, and reimplantation of new leads, but the long-term efficacy of this approach is unknown. The other one is balloon dilatation of the vein with stent placement [1]. Adjuvant anticoagulation is usually used after angioplasty. In our case, the patient refused the endovascular treatment so the medical approach was adopted. Furthermore, due to the long persistence of symptoms, we speculated that the thrombosis was not acute, and hypothesized that anticoagulation should restore a favorable balance between thrombosis persistence and physiologic fibrinolysis, leading to thrombus resolution. The anticoagulation treatment of upper extremity deep vein thrombosis (UE-DVT) has not been standardized yet; however, the current practice is to start warfarin after 5 to 7 days of low-molecular-weight heparin. Currently, there are no completed randomized trials of direct oral anticoagulants (DOACs) in UE-DVT; nevertheless, DOACs are increasingly used in real-world experiences with an adequate profile of efficacy and safety [6]. Thus, treatment of iatrogenic superior vena cava thrombosis with a DOAC represents an interesting new approach, to be validated in prospective trials against the current standard (heparin followed by warfarin).

## Data Availability

Not applicable.

## Abbreviations

SVC: Superior Vena Cava
CT: computed tomography
UE-DVT: upper extremity deep vein thrombosis
DOACs: direct oral anticoagulants
